# Gallbladder Duplication Associated with Gastro-Intestinal Atresia

**Published:** 2016-04-10

**Authors:** Rahul Gupta, Shilpi Gupta, Pramila Sharma, Anu Bhandari, Arun Kumar Gupta, Praveen Mathur

**Affiliations:** 1 Department of Paediatric Surgery, SMS Medical College; Jaipur 302004, Rajasthan, India; 2 Department of Radiodiagnosis, SMS Medical College; Jaipur 302004, Rajasthan, India

**Keywords:** Duodenal atresia, Gallbladder duplication, Atresia, Pyloric atresia

## Abstract

Gallbladder duplication in association with other GIT anomalies is a rare entity. We report two neonates; one with duodenal atresia and the other newborn with pyloric atresia, ileal atresia and colonic atresia, both were associated with gallbladder duplication which has not been reported earlier.

## INTRODUCTION

Gallbladder duplication is a rare anomaly occurring in about 1/4000 births. [1] Only one case of gallbladder duplication with associated fetal anomaly has been previously described in the English literature till date; the neonate had gallbladder duplication associated with type 3 duodenal atresia. [2] In our study, one neonate presented with duodenal atresia associated with gallbladder duplication; while the other newborn had gallbladder duplication associated with pyloric atresia, ileal atresia and colonic atresia. We herein, propose that there exists a common etiology for these associations.

## CASE REPORT

**Case 1:**

 A 12-day-old, preterm male neonate, weighing 1.9 kg, 1st in birth order, born to non-consanguineous parents, presented to paediatric department of our institute with non- bilious vomiting and decreased oral intake. There was no delayed passage of meconium but patient did not pass stool for last 48 hours. Antenatal ultrasounds were not done. On examination, child was hemodynamically stable, anicteric, mildly dehydrated, pulse rate-132/min and respiratory rate-56/min. Abdominal signs included mild epigastric distension, soft on palpation and absent bowel sounds; nasogastric aspirate was non-bilious. Laboratory values revealed an Hb of 19.1gm% and total leukocyte count of 14,300/mm3. Liver function tests, renal functions and serum electrolytes were normal. C-reactive protein was raised, and treatment started on the lines of necrotizing enterocolitis. Abdominal ultrasonography (USG) was not conclusive. Paediatric surgical opinion was sought and upper gastro-intestinal contrast study was performed which suggested duodenal atresia (Fig.1). 

**Figure:**
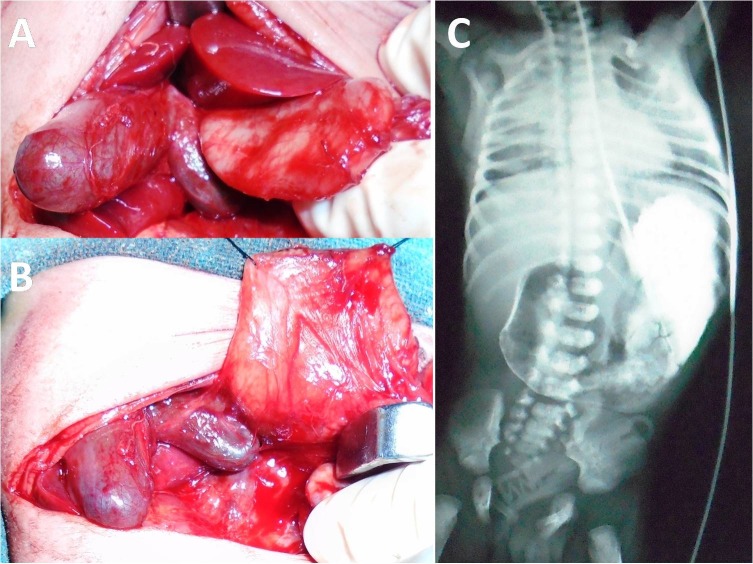
Figure 1: Intra-operative picture showing duplication of gallbladder with duodenal atresia (A); duodenotomy with stay sutures in place along with duplication of gallbladder (B); upper gastrointestinal contrast study showing complete duodenal obstruction with absence of air distally (C).

The child was taken up for laparotomy. Classic type 3 duodenal atresia was noted.The atresia was pre-ampullary. This was associated with a double gallbladder with a common neck as shown in Fig.1. No other anomalies were detected. Kimura’s diamond shaped duodeno-duodenostomy was done. Since there was no problem in biliary drainage, cholecystectomy was not considered. The post operative course was uneventful. Child was started oral feeds on post operative day 7 and discharged on post operative day 10.

**Case 2:**

A 2-day-old, preterm male neonate, weighing 1.6 kg, born by vaginal delivery was presented to our department with copious, non-bilious vomiting and epigastric fullness since birth. There was history of polyhydramnios on antenatal ultrasonography. On examination, neonate was hemodynamically stable, pulse rate-152/min and respiratory rate-50/min. The abdomen was soft and non-distended. Laboratory investigations were normal. An abdominal X-ray showed a single gas bubble suggestive of distended stomach with no air distally in intestine; contrast study of abdomen revealed non-passage of contrast beyond stomach. A presumptive diagnosis of pyloric atresia was made taking into consideration the history of antenatal polyhydramnios and X-ray findings. Laparotomy revealed distal ileal atresia associated with a double gallbladder with a common neck and pyloric membrane (Type 1 pyloric atresia) having a small central hole (Fig.2). The patency of the intestines distal to ileal atresia was checked by injecting saline through a no. 6 infant feeding tube which revealed membrane/web in transverse colon (colonic atresia). Excision of the pyloric membrane with Heineke-Mikulicz pyloroplasty, excision of colonic web and ileostomy with distal mucus fistula was performed. Cholecystectomy was not contemplated in view of major surgical procedure. Postoperatively patient had continued downhill course, developed sepsis and sclerema and died on 3rdpost operative day.

**Figure:**
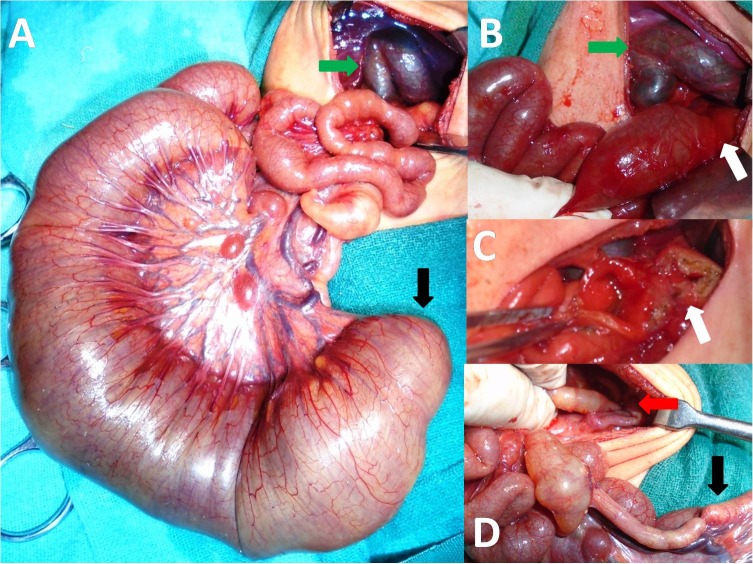
Figure 2: Intra-operative picture showing duplication of gallbladder (green arrow) with ileal atresia (A); pyloric atresia (white arrow) and gallbladder duplication (green arrow) (B); Heineke–Mickulicz pyloroplasty (C); colonic atresia (red arrow) and terminal ileum (black arrow) (D).

## DISCUSSION

The first reported human case of gallbladder duplication was noted in a sacrificial victim of Emperor Augustus in 31 BC. [3] Boyden’s classified gallbladder duplication into:(1) vesica fellea divisa (bilobed or bifid gallbladder, double gallbladder with a common neck); (2) vesica fellea duplex (double gallbladder with twocystic ducts), (a) Y-shaped type (the two cystic ducts uniting before entering the common bile duct) and (b) H-shaped type (ductular type, the two cystic ducts entering separatelyinto the biliary tree). [1,4]The exact pathogenesis of this condition is unclear and various theories proposed include. [2, 3] incomplete re-vacuolization theory, resulting in a persistent longitudinal septum that divides the gall bladder lengthwise; and occurrence of two separate cystic buds.

Only one case of gallbladder duplication associated with duodenal atresia has been described previously along with the present case. Also we report the index case of gallbladder duplication associated with pyloric atresia along with ileal atresia and colonic atresia which qualifies for Hereditary multiple intestinal atresias of the gastrointestinal tract.[5] Boyden et al. described the second part of duodenum as the “embryological traffic jam”.[6] Epithelial proliferation followed by occlusion (5thto 6th week) and failure of recanalization and re-vacuolization is the proposed embryogenesis of duodenal atresia; with second part of duodenum recanalizes at the end. [7] Boyden also suggested the relation between duodenal atresia and associated biliary ductal anomaly.[6] We also propose that there exists a common etiology (failure of recanalization) for association of duodenal atresia with gallbladder duplication.

Ultrasonographic appearance of gallbladder duplication may be mistaken for other common diagnoses like choledochal cyst, gall bladder diverticulum, Phrygian cap, extrinsic fibrous bands across the gallbladder, and a folded gallbladder of Horattas. [8]However in our case ultrasonography did not pick the anomaly and final diagnosis was made intraoperatively.

Though the literature on gall bladder duplications and previous report suggest that cholecystectomy could have been contemplated to prevent future complications, but we disagree with this view, as studies in paediatric patients are lacking and extrapolating the adult data would not be justified. [2]

## Footnotes

**Source of Support:** Nil

**Conflict of Interest:** None
